# Strain-specific surface polysaccharides mediate bacterial induction of metamorphosis in the coral *Pocillopora acuta*

**DOI:** 10.1093/pnasnexus/pgag159

**Published:** 2026-05-08

**Authors:** Marnie L Freckelton, Brian T Nedved, Michael G Hadfield

**Affiliations:** Kewalo Marine Laboratory, University of Hawaiʻi, 41 Ahui Street, Honolulu, Hawaiʻi 96813, USA; Kewalo Marine Laboratory, University of Hawaiʻi, 41 Ahui Street, Honolulu, Hawaiʻi 96813, USA; Kewalo Marine Laboratory, University of Hawaiʻi, 41 Ahui Street, Honolulu, Hawaiʻi 96813, USA

**Keywords:** *Pocillopora acuta*, metamorphosis, polysaccharide, biofilm, coral

## Abstract

Microbial biofilms induce larval settlement and metamorphosis in marine invertebrates, including corals, but the specific molecular cues for corals remain unclear. Glycosylated lipids and polysaccharides from crustose coralline algae, along with bacterial small molecules, are proposed inducers, but their effects vary by coral species, bacterial strain, and environment, highlighting the need to clarify their molecular basis and specificity. Here, we identify bacterial lipopolysaccharide (LPS), specifically its O-antigen polysaccharide component, as a settlement cue for larvae of the coral *Pocillopora acuta*. Using purified LPS from diverse marine bacteria, we show that inductive activity is bacterial species and strain specific and correlates with the structure of O-antigen. These findings support a glycan-centered mechanism of coral recruitment, implicating microbial surface chemistry as a key determinant of larval settlement. By demonstrating a specific and functional role for O-antigen, we expand the ecological relevance of bacterial glycans beyond immunity and pathogenesis, highlighting their role in key developmental and ecological transitions in marine invertebrates. As coral reefs face escalating environmental pressures, employing such signals offers a promising strategy to support reef restoration.

Significance statement The health and persistence of coral reefs depend on both the survival of adult colonies and their replenishment through the successful recruitment of new individuals, a process that is initiated when free-swimming coral larvae are triggered to settle, attach, and metamorphose in response to habitat-specific, surface-bound substances. The nature of these substances and their sources are active areas of research. Here, we show that the polysaccharide portion of the lipopolysaccharide outer coat of select biofilm-dwelling bacterial species triggers metamorphosis in a widely distributed Indo-Pacific coral. This finding points to a potential convergence between algal and microbial settlement cues at the level of surface-bound polysaccharides.

## Introduction

Most marine invertebrates begin life as planktonic larvae that must undergo settlement and metamorphosis to become benthic adults (1–4). Metamorphosis is rapid and usually irreversible, often unfolding within minutes to hours once specific environmental and biochemical conditions are encountered; it results in a permanent transition to a benthic lifestyle (1–3). The outcome of this event determines not only the fate of individual larvae but can also influence the patterns of population genetic structure and species distribution (2). As such, identifying the molecular nature of these settlement cues is essential for understanding larval recruitment and for designing tools to enhance settlement in reef restoration efforts.

Corals are ecologically important species that have been the focus of significant efforts to identify cues for settlement and metamorphosis. Much of this research has focused on acroporid corals and their interactions with crustose coralline algae (CCA), particularly *Porolithon onkodes* and *Hydrolithon boergesenii* (5, 6). These CCA species are widely regarded as potent inducers of settlement (6, 7). Numerous studies have shown that larvae of *Acropora* spp. reliably respond to live CCA and its biochemical extracts, including ethanol- and water-soluble fractions. This suggests that specific algal morphogens play a key role in initiating metamorphosis (5, 7–10). For example, Tebben et al. (8) identified glycoglycerolipids and unresolved polysaccharides in CCA fractions that induced settlement and metamorphosis in the larvae of *Acropora (tenuis) kenti*. However, CCA metabolomes vary substantially among species and with environmental conditions, which likely contributes to context-dependent differences in their settlement-inducing activity (11, 12). More recently, findings from Whitman et al. (7) have challenged the assumption that CCA universally induces settlement across coral taxa. Specifically, their study showed that live fragments of *Por. onkodes* triggered strong responses in larvae of *Acropora* spp., while larvae of many nonacroporid corals responded weakly or not at all. Taken together, these findings suggest that settlement responses attributed to CCA may not arise solely from algal metabolites.

Alongside research on CCA metabolites, other studies show that the settlement- and metamorphosis-inducing activity of live CCAs can be influenced by the microbial biofilms associated with them (13–16). For example, a study by Diaz-Pulido et al. (11) reported that coral settlement on living CCA correlated with elevated levels of specific di- and tri-saccharides. However, the source of the carbohydrates was unclear: the CCA itself, the associated biofilm, or interactions between them. Such difficulties have motivated efforts to identify specific members of CCA-associated microbial communities that contribute to settlement induction (17–19). Several corals respond to biofilm bacteria in the absence of CCA, including *Acropora microphthalma* (20)*, Acropora millepora* (9)*, Acropora palmata*, *Orbicella franksi*, *Porites asteroides* (19, 21)*, Pocillopora (damicornis) acuta* (22), and *Leptastrea purpurea* (17). In this context, isolates of *Pseudoalteromonas* spp. recovered from CCA-associated biofilms have yielded two chemically defined settlement cues: tetrabromopyrrole (TBP) (18) and cycloprodigiosin (23). TBP and cycloprodigiosin have been reported to trigger metamorphosis in multiple coral species (14, 16, 19). However, the ecological relevance of TBP remains uncertain because of its low abundance in biofilms and frequent failure to promote larval attachment, suggesting a partial or context-dependent role (8). Importantly, inductive bacterial isolates have also been recovered from marine biofilms lacking any association with CCA, demonstrating that settlement-promoting microbial cues extend beyond CCA habitats and may be widespread among benthic surfaces (20, 22, 24).

Outside of coral taxa, bacterial biofilm-induced metamorphosis has been demonstrated in representative taxa from every major marine invertebrate phyla with pelagic larvae, including mollusks, annelids, echinoderms, crustaceans, and sponges (1, 4). In the course of exploring potential settlement sites, coral larvae, like all marine larvae, encounter diverse microbial biofilms (1, 4). These bacterial biofilms form rapidly on all submerged surface, abiotic and biotic, and their composition varies with substrate type and local environmental conditions (4). This variation generates spatially and temporally dynamic chemical landscapes with the potential to signal the suitability of an environment for continued growth and development (25–27). Insights into the molecular mechanisms behind bacterial induction of settlement and metamorphosis in corals may benefit from other invertebrate model systems such as the polychaete tubeworm, *Hydroides elegans*. A cosmopolitan member of the biofouling community, *H. elegans* can be induced to settle and metamorphose by some, but not all, biofilm bacteria (28–30) and will not settle without the presence of bacteria (31, 32). It was reported that lipopolysaccharide (LPS), specifically its polysaccharide (O-antigen) component, is responsible for inducing metamorphosis in larvae of *H. elegans* in response to the biofilm bacterium *Cellulophaga lytica* (33). LPS, which is produced by Gram-negative bacteria and located in the outer leaflet of the outer membrane, exhibits structural variability that is influenced by both bacterial taxonomy and environmental conditions, making it a promising candidate for a broadly relevant yet environmentally tunable metamorphic cue. This result is in keeping with an emerging body of research that supports the hypothesis that surface-associated glycans represent a shared biochemical motif among settlement-inducing cues for marine invertebrate larvae from both algal and bacterial sources (8, 33–35).

Notably, some of the same bacterial species shown to induce tubeworm metamorphosis are also found on coral reef substrates and have been reported to induce settlement in *P. acuta* (22). However, the inductive potential of their LPS has not been tested directly, and it remained unknown whether LPS alone can explain biofilm-mediated settlement in *P. acuta*. In this study, we evaluated whether LPS from coral reef–associated bacterial strains can induce metamorphosis in larvae of *P. acuta* in the absence of live biofilms or algal cues. We focused on biofilm bacterial species previously isolated from Hawaiian coral reefs and identified by Tran and Hadfield (22) to be inductive for larvae of *P. acuta*. Specifically, we asked (i) is purified LPS sufficient to trigger settlement; (ii) does the inductive capacity vary among LPSs from different bacterial strains; and (iii) do structural features of the O-antigen polysaccharide mediate the larval response. To address the third question, we used lectin-binding assays to characterize terminal sugar residues on each LPS. Lectins, widely used in other invertebrate systems, provide insight into glycan structure and potential larval recognition motifs (36, 37). To further test whether inductive activity depends on the polymeric structure of the O-antigen, we also exposed larvae to individual monosaccharides corresponding to lectin-binding residues. This allowed us to assess whether specific sugar identities alone are sufficient, or whether higher-order glycan structure is required for inducing settlement.

By linking known settlement cues across phyla and testing the role of LPS in *P. acuta*, this study advances our understanding of coral–microbe interactions and contributes to the broader recognition of surface-bound polysaccharides as conserved mediators of larval settlement. We hypothesized that LPS, particularly its structurally diverse O-antigen component, represents a conserved microbial cue capable of inducing settlement and metamorphosis in larvae from across a broad range of marine invertebrate phyla. The experimental evidence presented in this study supports this hypothesis, indicating that such a mechanism may be shared across taxa and suggesting the possibility of a fundamental, evolutionarily conserved pathway underlying larval recruitment in the seas (4). Building on this understanding, microbial tools leveraging such cues may be designed to enhance coral settlement, offering a biologically grounded approach to support reef restoration efforts (38).

## Results

### Bioactivity of monospecific biofilms

**Figure 1:**
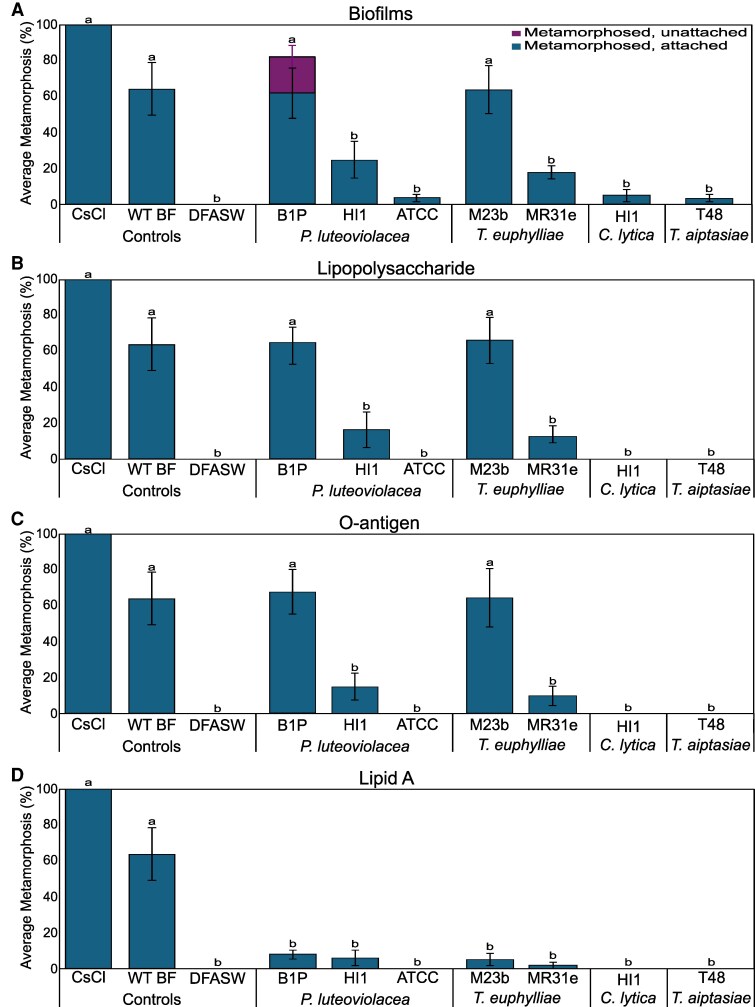
Settlement and metamorphosis in larvae of *P. acuta* after 24 h exposure to A) monospecific bacterial films, B) purified LPS, C) O-antigen, and D) lipid A. Results presented are for the most active concentration (5 µg/L). Wild-type biofilms (WT BF) and cesium chloride (5 mM CsCl) were used as positive controls, and sterile seawater (DFASW) was used for negative controls. Plots are averaged from three independent settlement bioassays. Error bars represent SE. Letters indicate significant differences in total metamorphosis calculated with Kruskal–Wallis and Dunn's test.

Larvae of *P. acuta* were induced to settle and metamorphose by monospecific biofilms from *Pseudoalteromonas luteoviolacea* B1P, but not from the strains HI1 and ATCC 33492. Similarly, larvae of *P. acuta* were induced to settle and metamorphose by monospecific biofilms from *Thalassotalea euphylliae* M23b, but not from *T. euphylliae* MR31e. Neither *C. lytica* HI1 nor *Tenacibaculum aiptasiae* T48 induced settlement and metamorphosis in the coral larvae (Fig. 1A). Metamorphosis without attachment was only observed for *Ps. luteoviolacea* B1P biofilms (Fig. 1A).

### Bioactivity of LPS extracts

Larvae of *P. acuta* were induced to settle and metamorphose by LPS (5 µg/L) extracted from *Ps. luteoviolacea* B1P, but not *Ps. luteoviolacea* HI1 or ATCC 33492 (Fig. 1B). Larvae of *P. acuta* were induced to settle and metamorphose by LPS extracted from *T. euphylliae* M23b, but not *T. euphylliae* MR31e. LPS extracted from *C. lytica* HI1 or *Te. aiptasiae* T48 was not inductive (Fig. 1B).

### Bioactivity of O-antigen and lipid A samples

*Pocillopora acuta* were induced to settle and metamorphose by isolated O-antigens from *Ps. luteoviolacea* B1P and *T. euphylliae* M23b only (Fig. 1C). No lipid A samples induced metamorphosis in these larvae (Fig. 1D).

### Impact of lectins on inductive activity of *T. euphylliae*

**Figure 2:**
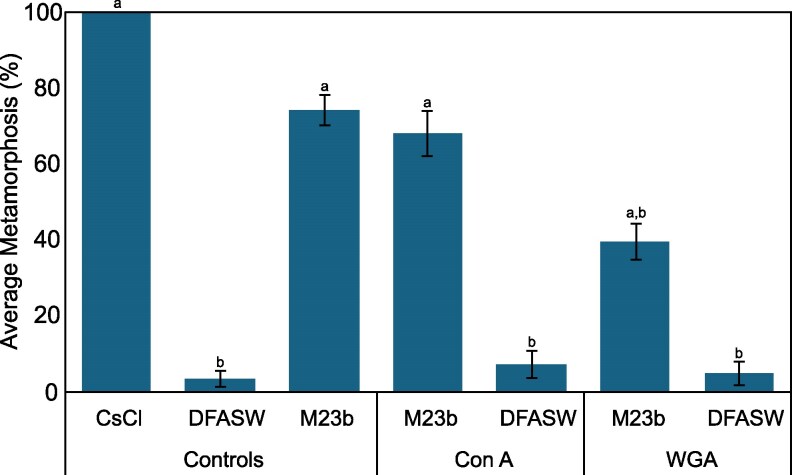
Settlement and metamorphosis in larvae of *P. acuta* after 24 h exposure to monospecific biofilms of *T. euphylliae* M23b with or without a 1-h preexposure with the lectins concanavalin A (ConA) or wheatgerm agglutinin (WGA). Positive larval control: cesium chloride (5 mM CsCl). Positive experimental control: biofilms of *T. euphylliae* M23b. Negative control: sterile seawater (DFASW). Plots are averaged from three independent settlement bioassays. Error bars represent SE. Letters indicate significant differences in total metamorphosis calculated with Kruskal–Wallis and Dunn's test.

The metamorphic inductive activity of *T. euphylliae* M23b was not inhibited by pretreatment with concanavalin A (Con A) but was partially inhibited by treatment with wheatgerm agglutinin (WGA; Fig. 2).

### Impact of monosaccharides on inductive activity of *T. euphylliae*

**Figure 3:**
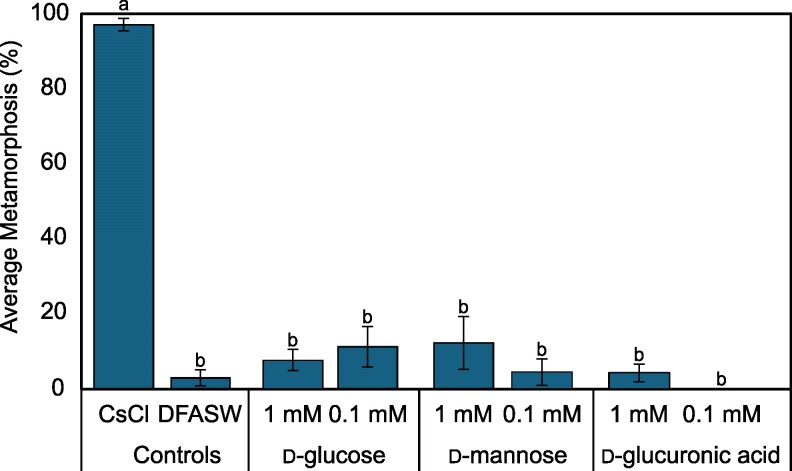
Settlement and metamorphosis in larvae of *P. acuta* after 24 h exposure to commercial monosaccharides (1 or 0.1 mM). Positive larval control: cesium chloride (5 mM CsCl). Negative control: sterile seawater (DFASW). Plots are averaged from three independent settlement bioassays. Error bars represent SE. Letters indicate significant differences in total metamorphosis calculated with Kruskal–Wallis and Dunn's test.

Individual monosaccharides did not recreate the activity of the isolated O-antigen or monospecific biofilms of *T. euphylliae* M23b (Fig. 3).

## Discussion

Here, we demonstrate that O-antigen from purified LPS derived from specific, inductive bacterial strains can directly induce metamorphosis in larvae from the coral *P. acuta*. This result aligns with prior demonstrations of LPS-mediated settlement in the serpulid tubeworm *H. elegans* (33). That the same class of molecules can induce these two organisms despite their deep evolutionary divergence is consistent with the idea that these molecules are either a conserved or repeatedly convergently evolved mechanism/cue regulating this pivotal life-history transition.

O-antigens are particularly well suited for a role in the induction of metamorphosis due to their immense structural complexity and variability (39–43). Unlike nucleic acids and proteins, which are synthesized via template-based mechanisms, glycan biosynthesis proceeds through nontemplate-driven enzymatic pathways, yielding branching architectures, variable monosaccharide compositions, diverse linkages, and anomeric configurations (39, 40). This resultant combinatorial potential, often referred to as the “glycan code,” enables highly specific biological messages to be encoded in glycan structure (39, 40). In the context of larval settlement, this modularity allows glycans to serve as environmentally and evolutionarily adaptable signals where subtle structural differences can yield distinct biological outcomes. This specificity is evident in our experimental findings: only a subset of O-antigen preparations induced metamorphosis in *P. acuta*, and induction was strain specific, even among bacteria of the same species. Furthermore, when inductive bacterial species were shared between the coral and tubeworm species, typically occupying different habitats, the inductive strains did not overlap.

That induction of metamorphosis by O-antigen differed across strains of the same bacterial species and also among invertebrate larval species suggests the potential ability of invertebrate larvae to discriminate among habitats based on fine-scale glycan structures, possibly via lectin-like receptors on the larval surfaces (36). This idea is supported by early experimental work on the polychaete *Janua (Dexiospira) brasiliensis*, where larval settlement and metamorphosis were shown to be inhibited by the monosaccharide D-glucose and by enzymatic treatments that disrupt glycoprotein structure, indicating a lectin-mediated recognition system between larval surface proteins and bacterial exopolysaccharides (36). In this hypothesis, lectins on the surface of the larva recognize and bind to glucose-containing sugar structures (glycoconjugates) found in the bacterial exopolymer. This binding triggers metamorphosis only when the shape and structure of the sugar molecules are compatible with the lectins, suggesting that a precise molecular match is required for the response to occur. Such a recognition system provides a plausible mechanism for larvae to interpret microbial cues as proxies for habitat suitability during substrate selection. This concept is not new; earlier studies have implicated lectin-mediated recognition in substrate selection and metamorphosis in both corals and ascidians (44–48). This highlights a critical point: the ability of larvae to distinguish subtle glycan variants may underlie species-specific habitat selection.

Typically, for a metamorphic cue to be ecologically relevant, it must be presented in a spatially constrained and biologically interpretable manner (31). O-antigens fulfill this criterion because they are surface bound, stable, and localized to microbial biofilms, in contrast to diffusible, waterborne cues that provide ambiguous spatial information (31). This spatial localization ensures that larvae encounter cues only upon direct surface contact, allowing for fine-scale habitat discrimination and reducing the risk of inappropriate or premature metamorphosis (31, 49). Our observations show that O-antigen-induced metamorphosis in *P. acuta* is dose dependent, consistent with the estimated concentrations of LPS in natural biofilms (50). These findings support the ecological relevance of O-antigen as a substrate-linked metamorphic cue. Future work examining structure–activity relationships, particularly through selective modification or removal of O-antigen moieties, will help define the specific glycan features responsible for inductive activity.

O-antigen is not the first or only surface-bound glycan to be identified as inductive for metamorphosis in marine invertebrate larvae. Indeed, surface-bound glycans are emerging as a unifying class of metamorphic inducers across diverse marine invertebrates. A growing body of evidence shows that structurally complex, surface-associated glycans can act as potent and ecologically relevant cues for larval settlement in taxa as diverse as cnidarians (8, 10, 51, 52), annelids (33, 50), and mollusks (35, 53, 54). For example, bacterial exopolysaccharides, such as Rha-Man and curdlan, induce settlement in the commensal hydrozoan *Hydractinia echinata* (51), while in *acroporid* corals, glycosylated lipids, and polysaccharides from CCA function as settlement signals (8, 10, 52). These chemically distinct but functionally similar molecules highlight a shared biochemical motif: persistent, substrate-linked glycans that facilitate species-specific recognition of suitable habitats. This specificity supports the notion of a modular, evolutionarily adaptable system that enables larvae to interpret complex landscapes during substrate selection.

In natural reef environments, coral larvae are unlikely to encounter monospecific bacterial biofilms or CCA devoid of a microbial coat. Instead, they interact with chemically complex, multispecies biofilms shaped by substrate type, hydrodynamics, and environmental gradients (1, 4, 55). Biofilms that develop on CCA or macroalgae differ markedly in microbial composition and metabolic output from those on bare rock or rubble (55–58). Even among CCA species, inductive and noninductive biofilms support distinct microbial communities (59). This heterogeneity creates a chemically complex and temporally dynamic landscape that settling larvae encounter. Its ecological complexity underscores the importance of considering settlement cues not in isolation but as part of a broader, multimodal sensory environment (7). This hypothesis aligns with broader patterns of cue synergy observed in marine invertebrate settlement and provides a mechanistic explanation for the variable effects of cues across taxa and conditions.

This study provides insight into how larvae interpret complex microbial landscapes. They further point to the evolutionary recruitment of bacterial surface glycans as reliable indicators of habitat suitability. Understanding the molecular basis of glycan-mediated signaling opens the door to targeted reef restoration strategies. It should be noted, however, that the very molecular features that make surface-bound glycans ideal cue molecules, their variability, and, in the case of O-antigen, their sensitivity to environmental conditions, also signal the vulnerability of these interactions to environmental disruptions. Anthropogenic stressors such as ocean warming (60–62), acidification (62–64), and pollution (65, 66) threaten the stability of microbial communities that produce inductive cues. Altered microbial communities can shift the availability of inductive taxa and the molecules they produce (4, 67, 68) and lead to very different eukaryote communities that develop upon them (69, 70).

Despite identifying LPS as a potent cue, important questions remain. The larval receptor systems that detect LPS and the precise moieties within O-antigens responsible for activity remain to be resolved. The observed variability in cue effectiveness among closely related bacterial strains reinforces the understanding that larval sensory systems are finely tuned to discriminate among glycans at high resolution. Future studies should investigate how O-antigens interact with other known cues—such as algal-derived glycans and small hydrophobic bacterial metabolites—and whether these cues act synergistically, additively, or hierarchically in driving settlement. Comparative analyses across coral species and in situ validations will be essential to reveal the full scope of glycan-based settlement mechanisms.

## Materials and methods

### Collection and culture of larvae from *P. acuta*

Adult colonies (10 cm) of the brooding coral *P. acuta* were collected from patch reef 11 in Kāneʻohe Bay, Hawaiʻi (21.43°N, 157.79°W) under permit from the State of Hawaii Division of Aquatic Resources. Colonies were collected at depths 1–3 m and transported to Kewalo Marine Laboratory within 1 h of collection in insulated containers filled with ambient seawater. In the laboratory, colonies were maintained in flow-through sea tables supplied with natural seawater (28 °C) and exposed to sunlight consistent with precollection conditions. Historically both *P. damicornis* and *P. acuta,* two species that have been difficult to distinguish morphologically (71), have been identified within Kāneʻohe Bay, Hawaiʻi. To ensure that the collected corals for our investigation were solely *P. acuta*, we sequenced the hypervariable mitochondrial open reading frame (ORF) to genotype all colonies according to the method of Flot et al. (72).

Larvae of *P. acuta* were collected during the peak release around the full moon in July and August. To collect larvae, adult colonies were placed into individual tanks (3 L) equipped so that seawater flowed through the tanks and into filter cups with 180 µm mesh, placed in bowls to maintain water levels above the mesh. Larvae were collected each morning within 2 h of release and transferred to clean containers with filtered (0.22 µm) seawater at a concentration of 1 larva/mL. Larvae were cultured at 28 °C with daily water changes and a 12-h light–dark cycle (180 µmol/photons m^2^ s^1^) using full spectrum light-emitting diode lights. The number of larvae released per colony ranged from 10 to 200 per brood. Larvae from multiple parent colonies were pooled prior to experiments to ensure sufficient larval numbers and to minimize parent-specific effects. All pooled larvae were considered to be the same age as they were released on the same day. Replicate experiments were performed on larvae from three separate full moon collections.

### Bacterial culture

Gram-negative bacteria (Table 1) were streaked from −80 °C glycerol stocks onto half seawater tryptone agar (1/2FSWt) (33) and incubated at 25 °C for 24–48 h. Single colonies were used to inoculate 3 mL broth cultures and incubated for 4 h at 28 °C with shaking (170 rpm). These starter cultures were adjusted to an OD_600_ of 1.000, and aliquots were used to inoculate overnight cultures. Cells were harvested by centrifugation (4,000 × *g*, 30 min, 4 °C) and washed with 1/10th volume of sterile seawater. All sterile seawater utilized in this study was double filtered (0.22 µm) and autoclaved (DFASW). Bacterial cells were then either frozen for later LPS extraction or diluted for biofilm formation.

**Table 1:** Species and strain identifications for bacterial isolates used in this study.

Species	Strain	Isolated from	Reference
*Cellulophaga lytica*	HI1	Pearl Harbor, Hawaiʻi	(30, 73)
*Pseudoalteromonas luteoviolacea*	H1	Pearl Harbor, Hawaiʻi	(30, 74)
	ATCC 33492	Seawater, France	(75, 76)
	B1P	Kāneʻohe Bay, Hawaiʻi	(22)
*Thalassotalea euphylliae*	MR31e	Kāneʻohe Bay, Hawaiʻi	(22)
	M23b	Kāneʻohe Bay, Hawaiʻi	(22)
*Tenacibaculum aiptasiae*	T48	Pearl Harbor, Hawaiʻi	(33)

### Monospecific biofilms

Washed bacterial cells were resuspended in sterile seawater and adjusted to produce a cell density of 10^8^ cells/mL for all strains (33). Adjusted bacterial solutions were added to 24-well plates and incubated to enable the attachment of cells. After 1 h, biofilmed wells were gently washed three times with sterile seawater to remove unattached cells. Biofilms were then ready for settlement assays.

### LPS extraction and purification

LPSs were extracted using Apicella's modification of the hot phenol method (77, 78) as previously described (33). LPS extracts were purified by adding 50% aqueous trichloroacetic acid (CCl_3_CO_2_H) at 4 °C to precipitate proteins and nucleic acids (77, 78). The supernatant was then dialyzed against distilled water and lyophilized to yield the corresponding LPSs. All LPS samples were checked for contaminating proteins, nucleic acids, and other lipid classes, as previously described (33). Each LPS fraction was assessed for metamorphic induction in larvae of *H. elegans* at 5 and 10 µg/mL.

### Separation of O-antigen and lipid A components

O-antigens were separated from lipid A components through mild acid hydrolysis of the LPSs with 2% aqueous acetic acid at 100 °C until lipid precipitation (6 h). The precipitate was pelleted by centrifugation (13,000 × *g*, 20 min), and the resulting supernatant was collected and lyophilized for use in settlement and metamorphosis assays. Spectrographic analysis confirmed that O-antigen samples were free of contaminating lipids and lipid A samples were free of contaminating polysaccharides (33, 79).

### Settlement assays

Settlement assays with larvae of *P. acuta* were conducted in 12-well plates. Each replicate evaluated 10 larvae, with six replicates per assay. Assays were performed in three independent trials to ensure reproducibility. Larval competency was confirmed through the use of the artificial inducer 5 mM cesium chloride or wild biofilms as positive controls (22). Wild biofilms were generated by submerging aragonite discs (Ocean Wonders) in the flow-through tanks for over 1 month, allowing natural colonization and maturation of microbial and algal communities on the surface. Discs were cut into 1 cm^2^ pieces for testing. Sterile seawater (DFASW) was used as the negative control. Settlement and metamorphosis were counted after 12 and 24 h exposure to biofilms. Because settlement and metamorphosis can occur independently in coral larvae, metamorphosis was recorded separately from settlement (18). Larvae that metamorphosed at the air–water interface were classified as metamorphosed but unattached, while those that attached to the experimental surface and then metamorphosed were recorded as metamorphosed and attached.

### Commercial monosaccharides

The monosaccharides D-glucose, D-mannose, and D-glucuronic acid were acquired from Sigma Aldrich. Individual stock solutions of each monosaccharide were made (10 mM) in sterile seawater and diluted to experimental concentrations (1 and 0.1 mM) before addition of larvae (36). Larvae were then observed for settlement and metamorphosis as described above.

### Lectin treatments

To bind and inactivate any O-antigens present within the biofilms, lectins were applied. Monospecific biofilms of *T. euphylliae* M23b were prepared as above. Before larvae were added for settlement assays, replicate biofilms were treated with the lectins at 2 mg/mL, following the protocol described by Kirchman et al. (80). The lectins used were concanavalin A (Con A) and wheatgerm agglutinin (WGA). Con A is mannose binding, and WGA is *N*-acetylglucosamine and sialic acid binding. Both mannose and *N*-acetylglucosamine have been implicated in other marine invertebrate metamorphosis studies (8, 51, 81). Additional controls of sterile seawater with each lectin were included.

### Statistical analysis

All statistical analyses were performed in Graphpad Prism 9 for Windows (GraphPad Software, San Diego, CA, United States, www.graphpad.com). Significant differences (*P* < 0.05) were calculated for total metamorphosis using Kruskal–Wallis followed by Dunn's test for multiple comparisons with false detection rate correction (82).

## Data Availability

All data used in this manuscript are deposited in Figshare and are publicly available (DOI: 10.6084/m9.figshare.29856860).

## Preprints

This manuscript was posted on a preprint at 10.1101/2025.08.22.671858.
